# A critical overview of the recent resurgence of polio in Pakistan as a public health concern for millions of children

**DOI:** 10.1097/MS9.0000000000004266

**Published:** 2025-11-10

**Authors:** Waseem Sajjad, Ayesha Eman, Pakeezah Tabasum, Ihsan Qamar

**Affiliations:** a Department of Medicine, King Edward Medical University, Mayo Hospital, Lahore, Pakistan; b Department of Medicine, Peoples University of Medical and Health Sciences for Women, Nawabshah, Pakistan; c Department of Medicine, Spinghar University, Kabul, Afghanistan

**Keywords:** Pakistan, poliomyelitis, poliovirus, resurgence, vaccination

## Abstract

Pakistan is the second largest country in South Asia. Despite significant national efforts from both central and state governments, Pakistan is still one of only two countries with continuous transmission of the wild poliovirus and active cases of poliomyelitis rendering millions of children with threat of lifelong disability. The purpose of this Perspective is to highlight the impending public health threat, identify and highlight the root causes of failure of Pakistan to eradicate polio, and call for a collective effort to combat the resurgence and eradicate this crippling illness from the country. Certain populations, such as children in rural areas, are more vulnerable. Many families either lack awareness or believe false information about the vaccine. Although Pakistan has implemented a range of interventions, such as National Wide Vaccination Campaigns, which have helped in the past, there are still gaps in planning and follow-up. In conjunction with religious and political strife, the challenges of eradicating the virus have been further compounded by the impacts of COVID-19, widespread flooding, and recurring infectious disease outbreaks. Misconceptions about vaccines and attacks on vaccination teams serve as significant barriers in failure to achieve the goal of polio eradication. Inconsistent governance and weak accountability systems are equally contributing factors to the failure of Pakistan in eradication of polio. Considering the factors contributing to the resurgence, we provide recommendations to introduce and implement robust vaccination to achieve the goal of polio-free Pakistan.

## Introduction

Poliomyelitis is caused by a virus, poliovirus. In Pakistan, polio persists as a major threat to millions of children, highlighting the need for robust eradication efforts. It typically affects children of age 5 years or less. Polio became the most feared disease in early 20th century as it continued to affect hundreds of thousands of children every year. The virus is highly contagious and spreads easily through direct person-to-person contact. Infected individuals harbor the virus in their throat for 1–2 weeks and excrete it in their feces for approximately 3–6 weeks, even if they do not show any symptoms. It can be transmitted through saliva, respiratory droplets, and contaminated food or water. 70%–95% of the cases present with a self-limiting flu-like illness. One in 200 cases present with rapid onset of flaccid paralysis with a high risk of lifelong disability or death^[1]^. As there is no approved antiviral treatment for acute poliomyelitis, prevention is the key. Polio survivors have poor quality of life with paralytic deficits, chronic pain, respiratory issues, and psychological distress. Post-polio syndrome (PPS) is a disabling syndrome that occurs years after the initial diagnosis. PPS prevalence is estimated to be 20–60% among polio survivors and typical onset is at least 15 years after the initial polio attack^[2]^.

**HIGHLIGHTS**

- The recent resurgence of polio cases in 2024 signals a potential epidemic risk for millions of children, threatening to undo previous eradication gains.
- Children under 5 years are the most vulnerable, especially in communities with limited vaccination coverage, facing severe health risks and lifelong disabilities.
- Low vaccination rates, driven by hesitancy, logistical issues, and resource limitations, are major contributors to the outbreak and require urgent attention to improve immunization efforts.
- Socioeconomic challenges and cultural misconceptions about vaccines hinder polio prevention, making community education and engagement crucial.
- Addressing this public health threat demands swift, coordinated action among national and global health agencies to prevent an epidemic and safeguard future generations.

The diagnosis of acute paralytic attack is usually clear on presentation with an asymmetric motor flaccid paralysis and aseptic meningitis. The virus can be isolated from the nasopharynx of the infected individuals for the 5 days, and from feces for about 5 weeks after the symptom onset. Two types of vaccine are currently available. Inactivated or killed vaccine (Salk), which is administered intramuscularly; and live vaccine (Sabin), which is administered orally. Polio virus is found in two forms: wild poliovirus (WPV), the most common form, and the circulating vaccine-derived poliovirus (cVDPV). The incidence of cVDPVs has been increasing in recent years. cVDPV type 2 caused 959 cases globally in 2020. The cause of cVDPV is low immunization rates. So, the best way to combat this situation is to vaccinate the children. Polio eradication strategy aims to ensure high (more than 80%) immunization coverage of children in the first year of life, with three doses of oral polio vaccine (OPV) as part of routine immunization schedules. It cannot eradicate the disease, but it increases population immunity. Hence, incidence declines, making eradication feasible in Pakistan accounting for a significant portion of these cases^[3]^. Polio virus has three serotypes, and all current activity is associated with type 1. There has been no case caused by type 2 since October 1999 and by type 3 since November 2011^[4]^.

Given these challenges, our Perspective aims to identify and highlight the factors responsible for the persistence of polio in Pakistan, failure of Pakistan in successful immunization against polio despite being wiped out globally, and highlight the importance of adequate vaccination and improved eradication strategies. Our study contributes to the literature by warning about responsible factors and the impending epidemic of polio in the region due to exponential increase in cases. This work has been reported in compliance to TITAN guidelines^[5]^.

## Polio in Pakistan: an overview of the past and present

**Figure 1.**
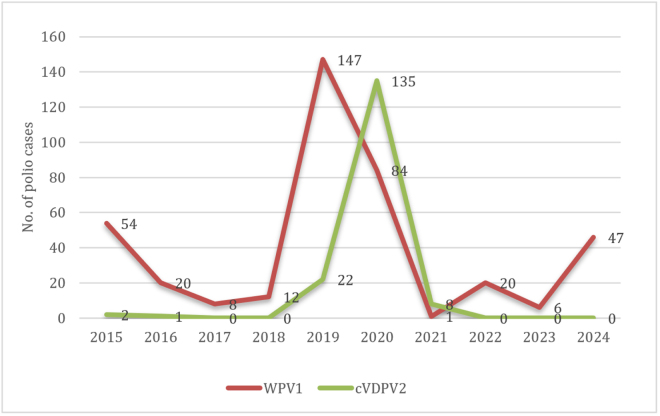
Cases of polio reported during the last 10 years (2015–November 2024).

Since the launch of Global Polio Eradication Initiative (GPEI), the global incidence of polio cases decreased from an estimated 350 000 in 1988 to just 407 in 2013^[6]^. Pakistan and Afghanistan are the only two countries in the world where polio cases are being reported since 2017. In Pakistan, polio vaccine was included in the Expanded Program of Immunization supported by the Global Alliance for Vaccine and Immunization. Pakistan’s polio eradication program began in 1994 with the launch of its national surveillance and the establishment of immunization teams made up of workers going door to door to administer OPV to children under 5 years^[6]^. Polio cases were significantly reduced till 2005, reporting non-polio-associated acute flaccid paralysis (AFP) rate increased from 1.53 to 3.53 cases per 100 000. However, this was followed by an increase in subsequent years. Pakistan’s geographical location is crucial in this context, as it shares an extensive border with Afghanistan, particularly along the Khyber Pakhtunkhwa and Balochistan areas, which are polio-endemic^[6]^. This volatile environment fueled suspicions that vaccination programs were part of a hidden agenda, further obstructing vaccination efforts in these areas^[5]^. Pakistan is a conservative society where religious beliefs play a big role. Armed militancy significantly hindered the polio vaccination campaign. Rumors of vaccines being a Western plot to sterilize children have fueled refusals^[7]^. The onset of the COVID-19 pandemic in Pakistan after February 2020 and the resulting lockdown led to the disturbance in the vaccination procedure and to an increase in the number of polio cases in Pakistan^[8]^. However, in the early 2021, vaccination campaigns resumed and a decrease in polio cases was reported^[8]^. In 2021, only one case of WPV was reported, bringing hope for eradication of the virus. However, after a 15-month break, the resurgence was noted when a case of WPV was identified in a child from North Waziristan^[9]^. Floods compounded the challenges faced by an already struggling country to eradicate polio. Figure 1 illustrates the trend in the number of cases over the last decade^[10]^.

## The recent resurgence

**Figure 2.**
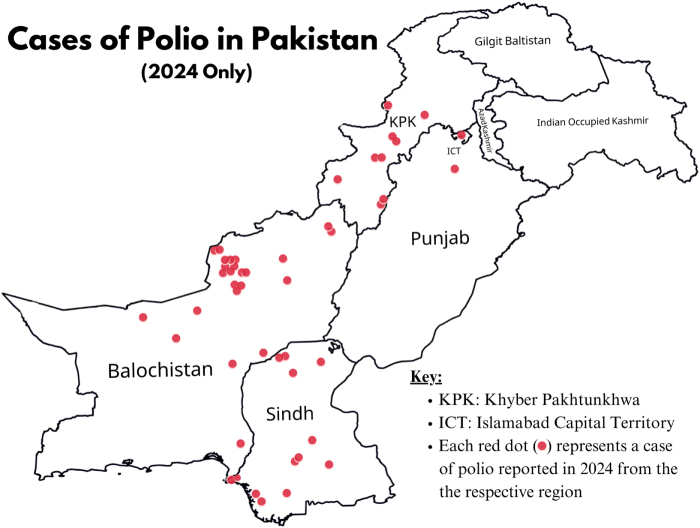
Representation of the provincial burden of polio cases in 2024.

On March 14, the first polio case of 2024 was reported from Dera Bugti, Balochistan, which raised many questions on the effectiveness of the measures taken and the efficiency of the campaigns being run in the country^[11]^. Before the detection of this case, two nationwide polio vaccination campaigns in 2024 had already been conducted, vaccinating more than 45.4 million children under 5 years against polio. This resurgence led to the acceleration of the efforts by case response system starting from March 26 in affected districts to prevent the further spread of disease^[11]^. The campaign conducted from March 25 to 28 vaccinated millions of children in polio-endemic areas, with special focus on KPK and Balochistan. On October 28, Pakistani officials announced the launch of a nationwide polio vaccination campaign to immunize over 45 million children under 5 years. In its statement, the program stressed the urgency of the situation, urging parents to recognize its seriousness and open their doors to vaccinators during the campaign. However, the chain of new cases continued unabated, with each new case adding to the growing toll. In contrast, India, once considered one of the toughest places to eradicate polio, successfully achieved eradication in 2014 by adopting multi-sectorial, community-engaged approach, including door-to-door vaccination campaigns, local influencers, and robust surveillance system that addressed both logical and socio-cultural barriers. Pakistan’s efforts, while extensive, have lagged this level of integration and local trust-building, indicating that the issue lies not in the scale of vaccination but in socio-political dynamics that influence its reception and effectiveness^[12]^. An independent monitoring board of the GPEI reported that over 420 000 children in Pakistan were not vaccinated during the 2024 anti-polio campaigns. The report revealed more than 200 boycotts in Khyber Pakhtunkhwa, highlighting that violence, insecurity, and resistance continue to be as widespread as in 2023. Communities often demanded reliable electricity before allowing vaccination^[11]^. The Regional Reference Laboratory for Polio Eradication at the National Institute of Health (NIH) has reported the detection of WPV type 1 (WPV1) in environmental (sewage) samples collected from 27 districts in Pakistan. As of 1 November 2024, WPV1 was found in sewage samples collected from 27 districts^[13]^. As of January 2025, Pakistan reported 71 WPV1 cases, with additional cases emerging in regions like Gilgit-Baltistan. Figure 2 represents the polio cases reported from different areas in 2024, indirectly highlighting the burden of the disease in the respective regions of Pakistan with Balochistan as the epicenter of 2024 cases^[13]^.

## Why Pakistan failed? Identifying vulnerable population and barriers to successful immunization

The persistence of the disease can be attributed to various factors operating at multiple levels. Weak government policies have inadequate vaccination initiatives, while limited community engagement and a conservative mindset among certain segments of the population prevent many from recognizing the urgent need for vaccination. Limited accessibility to certain areas, coordination challenges with local administration and security concerns are some of the major challenges. Poor healthcare system, coupled with difficult geography of Pakistan, serves as barrier to the vaccine delivery in mountain areas, placing the population at risk. For instance, nomadic communities in Balochistan, refugees, and migrant workers face unique access challenges. Poverty, illiteracy, lack of awareness, and false religious beliefs are especially common in areas near Pak-Afghan border. Certain sects believe that the vaccine contains ingredients prohibited by their religion, and some extremists propagate false information claiming that vaccination is a conspiracy or will cause sterilization in their children as it was a part of some Western agenda. This instills fear in parents, resulting in their refusal to vaccinate their children. Up to 23% of parents in high-risk districts have refused polio drops at least once according to a survey by UNICEF^[14]^. The US Central Intelligence Agency launched a fake vaccination campaign in Pakistan in 2011 to track Osama bin Laden which fueled the mistrust, further contributing to vaccine refusal by parents^[15]^. These barriers span policy, sociocultural, and logistical domains collectively hindering the success of polio immunization efforts in Pakistan.

**Figure 3.**
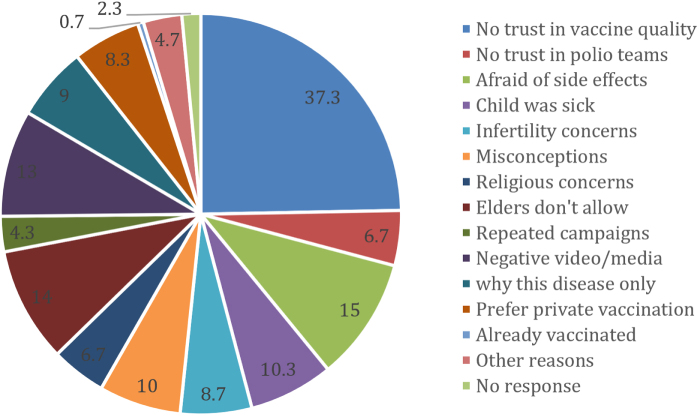
Causes of failure to successful immunization against polio in Pakistan.

The killing of polio workers in both Pakistan is of great concern. More than 200 polio team workers have lost their lives, including female workers, male workers, police, and security personnel, and a large number of casualties have also been reported^[16]^. On 16 January 2016, polio workers died in a suicide attack in Quetta, while six female polio workers were shot and killed in the same city^[16]^. Sixty-eight deaths were reported all across Pakistan from December 2012 to January 2014^[16]^. Alongside this, there have been multiple reports of physical and verbal abuse, as well as abduction of vaccination team workers^[16]^. These incidents collectively highlight a profound lack in public awareness, coupled with socioeconomic instability, fragile infrastructure, and suboptimal functioning of environmental surveillance systems. Together, these factors create a challenging sociopolitical climate that fuels the ongoing struggle against polio in Pakistan, exacerbating the crisis and hindering progress at every turn. Figure 3 displays 34 various factors by union councils of Karachi, Sindh, for vaccine refusal, highlighting no trust in vaccine quality as the leading refusal factor^[17]^.

## A fate or choice? Debate on Pakistan’s past attempts and current initiatives for polio eradication

In 1978, routine immunization against poliovirus and five other diseases was started, but special attention to polio vaccination was given in 1994, with evolving strategies and intensifying efforts of vaccinating maximum children in polio-endemic areas. National surveillance of AFP was also initiated in 1994^[18]^. In 2000, the strategy incorporated door-to-door visits with at least seven rounds of national immunization days to maximize the public involvement^[17]^. NIH located in Islamabad is a WHO-accredited poliovirus laboratory. Analysis of stool and sewage samples, AFP surveillance, and provision of genetic data on circulating polio viruses are some of the tasks performed by the NIH. Overall polio cases in Pakistan decreased significantly till 2014 by >93%, after which a rise in cases was reported. The Pakistan Polio Eradication Program is a big platform led by The Government of Pakistan, working in partnership with the WHO, UNICEFF, BMGF, and CDC, where the UNICEF supports community engagement to address hesitancy. The National Emergency Action Plan (NEAP) is the eradication strategy that was developed in 2020 with the goal to vaccinate all the children under 5 years, to ensure rapid detection of any new cases, and to develop both national and international strategies. Despite decades of strategic efforts led by the NEAP, polio remained endemic due to persistent implementation gaps, poor intersectoral coordination, weak accountability, and inadequate community engagement. Opportunities lie in integrating scholarship efforts with broader health services and expanding female vaccinator networks. But threats like vaccine hesitancy, security issues, and eroded public trust continue to hinder progress^[19–21]^. AFP surveillance detects any new cases, and environmental surveillance detects poliovirus samples in sewage or environmental samples. There are 127 environmental sampling sites working in 87 districts of Pakistan^[19]^. Several polio drives are launched across the country, particularly after the detection of new cases. Their success rates vary because vaccine delivery to challenging areas, vaccine hesitancy, and inadequate reporting systems continue to be the barriers and contribute to preventing the campaigns from achieving their goals. Despite the rise in cases at an alarming rate, there is a disturbing increase in attacks on polio vaccination workers^[16]^. On 1 November 2024, an attack on a police van escorting a polio vaccination team resulted in nine casualties, including five children and left several others injured^[22]^. Similar incidents are being reported in many other areas.

## Recommendations for polio eradication

A key strategy to eradicate the disease from the country is to bolster the mop-up campaign following the reemergence of the disease, closely monitor any new cases with a rapid response, and further intensify vaccination efforts in high-risk areas.

Provision of staff with adequate infrastructure including improved sanitation facilities and clean water supply, social mobilization including educating the public to dispel misconceptions and raise awareness about the disease and the benefits of vaccination, leveraging digital technology such as short video campaigns featuring trusted local figures to dispel myths, tracking processors to track the vaccinated and unvaccinated children, helping to tailor strategies based on the unique needs of each area, and security surveillance for polio workers to feel protected while carrying out their duties to all areas of the country are some of the basic steps toward eradication of the disease^[23]^. Prioritizing security for polio workers and community education is critical for immediate impact. Moreover, vaccination campaigns should ensure that every child under 5 years receives at least three doses of the OPV. Efforts must focus on educating the public to dispel misconceptions and raise awareness about the disease and the benefits of vaccination.

To mitigate the spread of contaminated water, strategies should be established for environmental surveillance to detect poliovirus strains excreted by individuals who have received the OPV. Local leaders in the tribal areas should be actively involved in this effort providing long-term actions. Given their influence and authority, these leaders should take the initiative to educate their communities to cooperate with the vaccination teams. Accurate reporting of new cases with early response is very important as timely action is crucial to halt the spread of the disease. The power of social media is undeniable today, and various organizations in the country should unite on a common platform. Seminars on polio eradication efforts should be conducted across all regions. Numerous student organizations and young doctors are already organizing health camps. With funding from authorities and security provided by the state, these efforts can reach new heights of success. These measures provide short-term support to the polio eradication goal, while measures like public awareness through caller tunes could prove beneficial providing long-term actions in collaboration with local administration, the government should launch more effective initiatives across the country with particular focus on polio-endemic areas. Door-to-door visits, counseling of parents regarding vaccine safety, and involvement of local heads seem to be very effective measures to deal with vaccine hesitancy. More robust data collection system should be implemented through community healthcare workers and school-based systems. A regular examination of epidemiological data with modest funding for training, community workers to enhance data accuracy, and the implementation of robust surveillance policies, along with the measures mentioned above, can help eradicate the virus.

## Conclusion

The significant surge in the number of polio cases in 2024 compared to the past raises numerous questions about the measures being implemented to combat the disease. Millions of children are at risk of contracting this disabling disease. This serves as an urgent call to action for both higher authorities and local administration. If one country has a circulating virus, the entire globe is at risk, as asymptomatic individuals can spread the virus without being easily identified. This situation places an even greater responsibility on Pakistan to reassess its strategies and develop a robust mop-up plan to combat the disease, for instance, deploying mobile vaccination units to high-risk districts like Balochistan. While challenges like vaccine hesitancy persist, sustained commitment to these measures can pave the way to a polio-free Pakistan.

## Data Availability

No datasets were generated for this study.
